# Emergency Endovascular Aneurysm Repair for Aortoduodenal Fistula After Interposition Tube Graft: Initial Damage Control Surgery at a Primary Care Hospital

**DOI:** 10.7759/cureus.104618

**Published:** 2026-03-03

**Authors:** Tomas Marin Cuartas, Kristina Lenz, Stefan Lenz, Jörg P Ritz, Carsten Rosenkranz

**Affiliations:** 1 General Surgery, Helios Kliniken Schwerin, Schwerin, DEU; 2 General Surgery, ELBMED Hospital Prignitz, Prignitz, DEU; 3 Vascular Surgery, Helios Kliniken Schwerin, Schwerin, DEU

**Keywords:** aortoduodenal fistula, damage control surgery, endovascular aneurysm repair, evar, multidisciplinary approach, upper gastrointestinal hemorrhage

## Abstract

Aortoenteric fistula (AEF) is an abnormal connection between the aorta and the adjacent gastrointestinal tract. Primary AEFs occur without previous aortic surgery, whereas secondary AEFs develop months to years after aortic procedures such as graft placement. Although rare, AEFs are a life-threatening cause of upper gastrointestinal bleeding that require prompt diagnosis and emergency management. Without timely treatment, the condition carries a high mortality rate. We present the case of a patient with massive upper gastrointestinal bleeding and hemorrhagic shock due to a secondary aortoduodenal fistula, occurring four years after placement of an interposition tube graft for an infrarenal aortic aneurysm. The patient was successfully managed through a multidisciplinary approach, combining endovascular aneurysm repair (EVAR) with open surgical intervention. This case highlights a rare and often overlooked cause of gastrointestinal hemorrhage in patients with a history of abdominal aortic surgery. The article discusses the pathophysiology, diagnosis, and management strategies for secondary AEFs.

## Introduction

Aortoenteric fistula (AEF) is a rare pathological communication between the aorta and the adjacent gastrointestinal tract, most commonly involving the third and fourth portions of the duodenum [1-4]. The first documented case of AEF was described by Cooper over 200 years ago, in 1822 [5]. Primary AEFs occur in patients without a history of aortic surgery and typically result from erosion of a dilated infrarenal aneurysm, an infected aortic wall, or an inflamed segment of the aorta into the posterior duodenal wall [6,7]. Although rare, secondary AEFs are more frequent and represent the most severe late complication of abdominal aortic reconstructive surgery. AEFs are a life-threatening cause of upper gastrointestinal bleeding and require prompt recognition and emergent intervention. Without timely treatment, the condition carries a near-100% mortality rate [1,8].

## Case presentation

A 64-year-old patient with a history of recurrent gastrointestinal bleeding due to duodenal angiodysplasia, treated twice endoscopically and with a prior open repair of an infrarenal aortic aneurysm using a tube graft four years earlier, presented to a primary care center with recurrent hematemesis and crampy abdominal pain. An initial gastroduodenoscopy revealed severe active bleeding from multiple Cameron ulcers in the proximal stomach (Forrest 1B) and a large paraesophageal hiatal hernia. The next day on follow-up endoscopy, a large duodenal ulcer in the distal duodenum was identified and treated with adrenaline injection and coverage using Purastat gel. However, a few hours later, the patient developed hemodynamic instability, prompting an emergency gastroscopy. This revealed a duodenal hemorrhage that was unresponsive to endoscopic intervention. Consequently, the general surgery team proceeded with emergency laparotomy. In retrospect, an aortoduodenal fistula was not initially suspected, likely due to anchoring bias related to the patient’s history of recurrent bleeding from angiodysplasias.

In the primary care center, the patient arrived in the operating room in hemorrhagic shock under an ongoing massive transfusion protocol. A median laparotomy was performed, followed by Kocher maneuver mobilization of the duodenum. Intraoperative exploration identified an AEF with a palpable aortic tube graft as the source of massive bleeding. In the absence of vascular surgery capabilities, urgent hemodynamic stabilization was prioritized to enable secondary transfer to a specialized tertiary center, following the principles of damage control surgery (DCS).

As part of the DCS approach, the mesenteric root was encircled with a vessel loop to aid in hemorrhage control, followed by complete mobilization of the horizontal segment of the duodenum. The duodenum was then transected distal to the duodenojejunal flexure using an Endo-GIA stapler, and the distal stump was closed. Proximally, a Foley catheter was antegradely inserted via a gastrostomy and balloon-inflated to temporarily occlude the duodenum and control hemorrhage near the fistula site. After achieving hemodynamic stabilization, temporary abdominal closure was performed using a modified Bogota bag technique with the Alexis® retractor system, preserving peritoneal domain and allowing safe re-entry. The patient was subsequently transferred via air ambulance to a tertiary care center for definitive vascular reconstruction.

**Figure 1:**
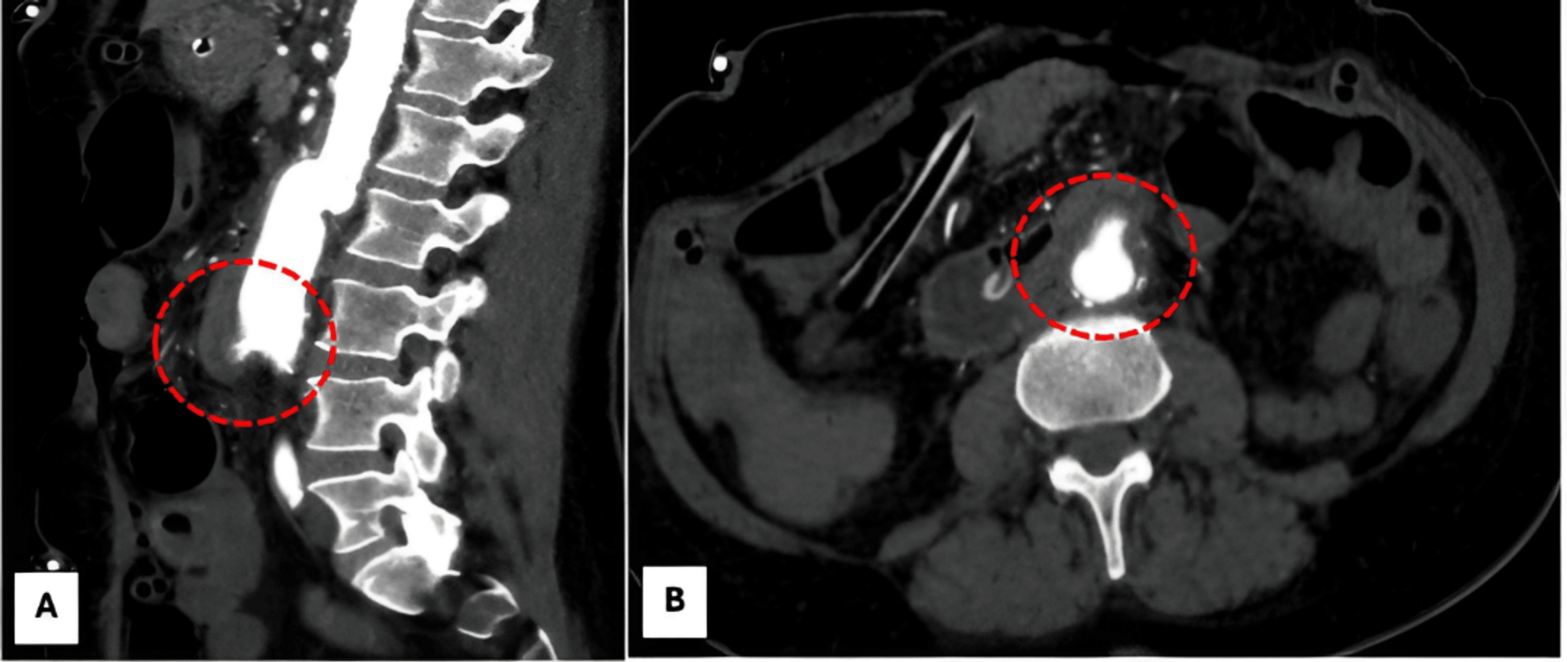
Extravasation of contrast material from the aorta into the intestinal lumen (dotted line). Panel A: sagittal view; Panel B: transverse view.

Upon arrival at the emergency department at the tertiary care center, the patient was hemodynamically stable, allowing for a computed tomography angiography of the aorta and pelvis to be performed. Imaging revealed an aneurysmal dilation of the abdominal aorta measuring 3.9 cm x 1.5 cm proximal to the aortic bifurcation. Moreover, a direct communication between the aorta and the duodenum was identified (Figure 1). These findings confirmed a contained rupture at the distal ventral anastomosis site of a previously implanted tube graft for an abdominal aortic aneurysm (AAA), with the formation of an aortoduodenal fistula.

Following confirmation of the diagnosis and given the open abdomen (abdomen apertum) and the need for urgent bleeding control, the decision was made to proceed with endovascular treatment. An aortobiiliac stent graft was implanted via bilateral transfemoral access using the MANTA vascular closure system. Control angiography confirmed patency of both renal arteries and revealed no evidence of endoleak.

**Figure 2:**
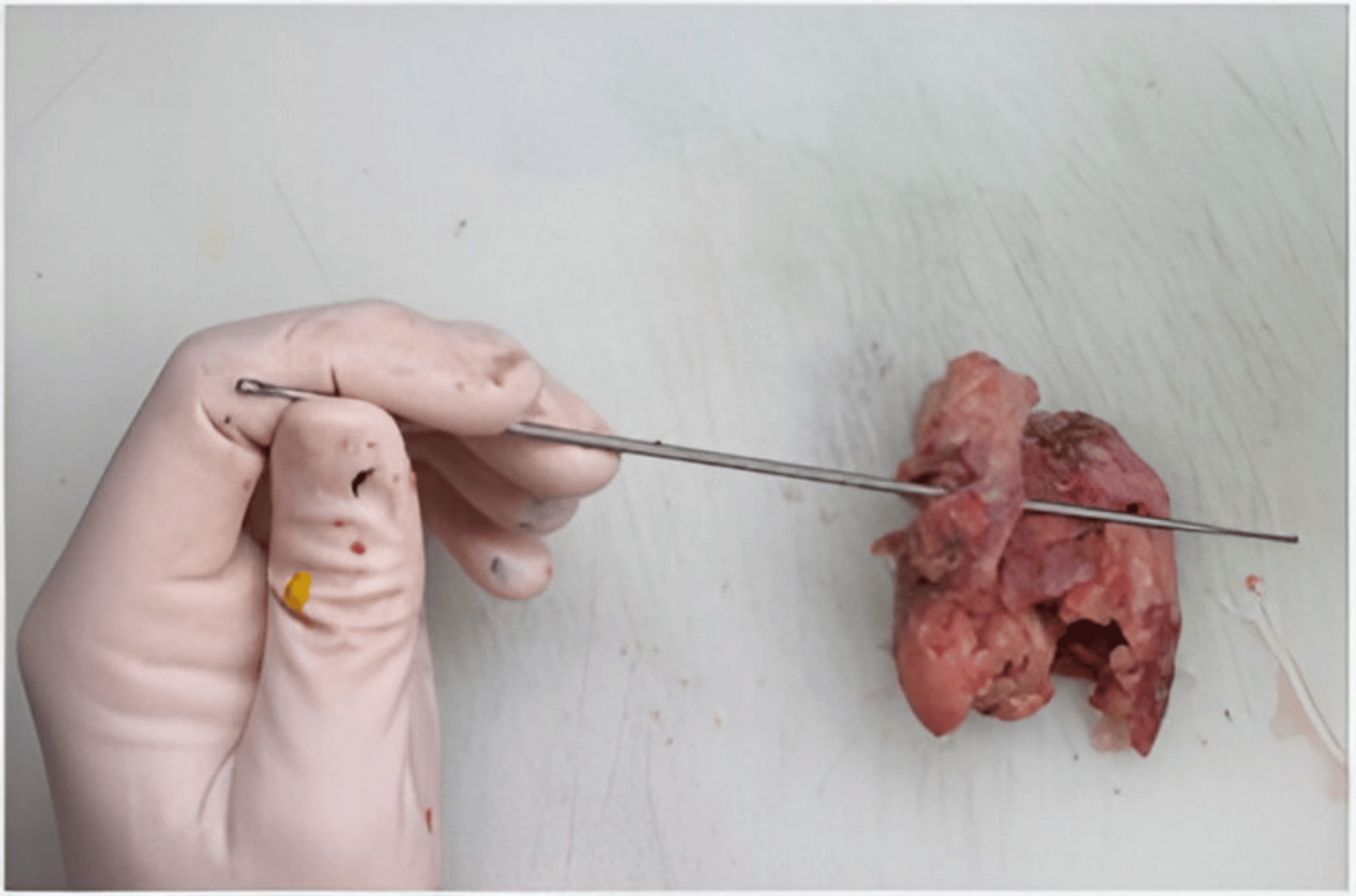
Fistulous defect identified in the intestinal mucosa, consistent with aortoenteric communication.

Subsequently, the patient underwent a laparotomy performed by the visceral surgery team. Intraoperatively, the duodenum was found to be firmly adherent to the aorta at its third (horizontal) portion. Careful and meticulous dissection enabled separation of the fistula-bearing duodenal segment from the aortic wall (Figure 2). Samples from the old tube graft were taken. The affected area was then covered with an omental flap, and intestinal continuity was restored through reanastomosis.

**Figure 3:**
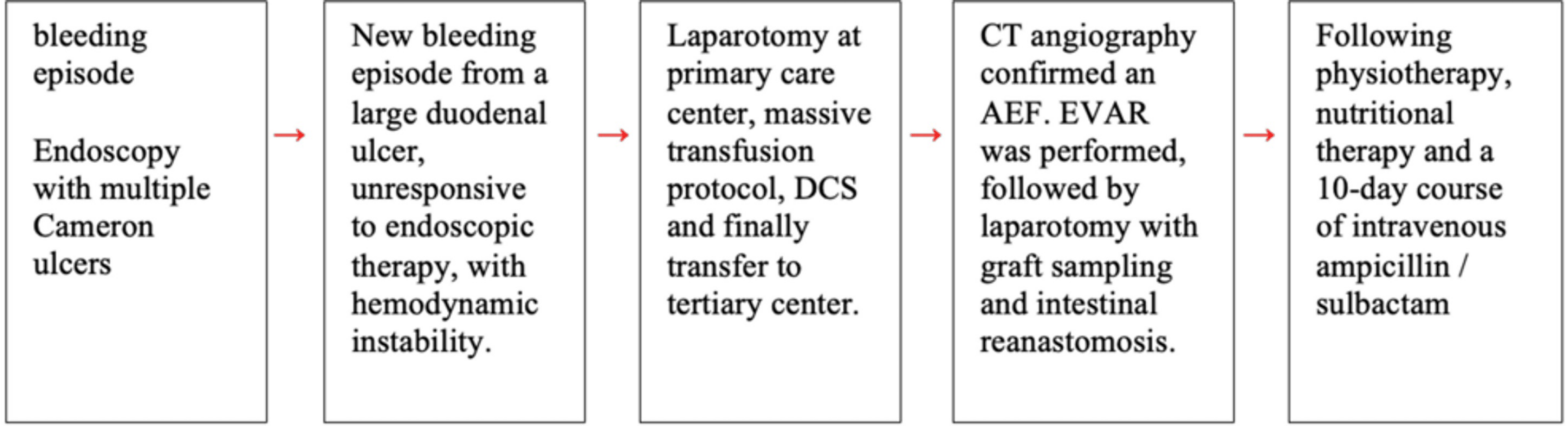
Timeline of clinical events DCS, damage control surgery; AEF, aortoenteric fistula; EVAR, endovascular aneurysm repair

The patient’s course in the intensive care unit was very favorable. Catecholamine support was gradually reduced, and the patient was successfully extubated. Blood cultures remained negative throughout hospitalization; however, intraoperative culture of the aortic graft grew multisensitive *Staphylococcus epidermidis*. Following physiotherapy, nutritional therapy, and a 10-day course of intravenous ampicillin/sulbactam, she was discharged on hospital day 11 with prescriptions for an additional four-week course of oral antibiotics, acetylsalicylic acid, and pantoprazole. At discharge, she was scheduled for strict outpatient follow-up with the vascular surgery team. A timeline of clinical events is presented in Figure 3.

## Discussion

AEF incidence is extremely low, with an annual rate of approximately 0.007 per million, representing around 0.04-0.07% of autopsy findings for primary AEFs and up to 1% for secondary AEFs [4,7,9]. The average age of presentation reported in the literature is between 63 and 64 years, with a marked male predominance (75-80%) [9].

Due to the close anatomical proximity of the infrarenal aorta to the distal duodenum, AEFs most commonly involve the duodenum, accounting for 75-93% of all cases, and the third portion is affected most frequently (69%), followed by the fourth portion (7%) [7,9].

The majority of primary AEFs are associated with atherosclerosis or aneurysmal disease, particularly the progressive expansion of an AAA, which leads to chronic mechanical pressure on the gastrointestinal tract, resulting in fibrosis and inflammatory tissue destruction [10]. Less commonly, primary AEFs may arise from infections, neoplasms, radiation-induced injury, ingested foreign bodies, or cystic medial necrosis [1,6,7,9].

Secondary AEFs are approximately 10 times more frequent than primary ones and typically occur in patients with a history of aortic prosthetic reconstruction [10]. Persistent transmission of pulsatile forces from the aorta to the adjacent bowel can result in erosion of the prosthetic graft into the gastrointestinal tract [8]. Additional contributing mechanisms include mechanical wear at the suture line, anastomotic disruption with pseudoaneurysm formation, and eventual fistulization [1,2,10].

These complications can occur within weeks to several years following the initial surgical intervention. While the occurrence of AEF after open AAA repair is well established, endovascular aneurysm repair (EVAR) was initially considered to carry a lower risk, owing to the preservation of the native aortic adventitia and the absence of direct anastomosis or suture lines [6]. However, multiple reports have documented cases of AEF following EVAR, and it has been suggested that EVAR may, in some instances, induce significant inflammation in the aorta and surrounding tissues, potentially contributing to fistula formation [11,12]. Notably, approximately 25% of patients with stent graft infections go on to develop AEFs, a complication associated with markedly poor prognosis [11].

The classic triad of gastrointestinal bleeding, abdominal pain, and a pulsatile abdominal mass is present in fewer than 25% of patients with primary AEFs [9,13].

The most common clinical manifestation of AEF is intermittent upper gastrointestinal bleeding, classically referred to as a "herald bleed." This may present as melena, hematochezia, or hematemesis and is typically self-limited. However, in approximately one-third of cases, it is followed within hours to days by a massive hemorrhage [4,6,7,9,10]. The intermittent nature of the bleeding is often due to temporary occlusion of the fistula by thrombus formation [7,9]. Patients may also present with non-specific symptoms such as recurrent fever. In some cases, AEFs can be asymptomatic and discovered incidentally during routine health evaluations, such as testing for occult blood in stool [8].

AEFs should be considered a potential cause of massive upper gastrointestinal bleeding in any patient with a history of abdominal or thoracic aortic aneurysm repair, particularly those who have undergone open surgical reconstruction or endovascular repair with a prosthetic graft [2,6,7].

Endoscopy, abdominal computed tomography, and angiography are valuable diagnostic tools in the evaluation of AEFs and in identifying alternative causes of upper gastrointestinal bleeding. Imaging plays a crucial role in detecting AAA, monitoring aneurysm growth, identifying complications such as rupture or fistula formation, and guiding both preoperative planning and postoperative surveillance. Among available modalities, CT has emerged as the gold standard for suspected AEF, with a reported sensitivity of up to 90% [3,4,11]. CT is less invasive than endoscopy or angiography, is more readily accessible, and does not carry the risk of thrombus dislodgement in the fistula [7,9].

Key CT findings suggestive of AEF include ectopic gas, loss of the normal fat plane between the aorta and adjacent bowel, extravasation of contrast material from the aorta into the intestinal lumen, focal intestinal wall thickening greater than 5 mm, and leakage of enteric contrast into the para-prosthetic space [3,4,7,8].

Endoscopy is a first-line diagnostic approach in the evaluation of acute gastrointestinal bleeding and remains essential for excluding other potential sources, such as gastroduodenal ulcers, esophageal lesions, or varices [7,9,10]. However, its sensitivity for diagnosing AEF is low, estimated at approximately 24% [3,8,11], and a negative result does not exclude the diagnosis. Endoscopic findings suggestive of secondary AEF may include visible fistulous tracts, active bleeding, or visualization of a prosthetic graft within or crossing the intestinal lumen [8].

Optimal management of secondary AEFs requires addressing both the aortic and enteric defects. The fundamental principle involves exclusion of the fistula. EVAR may serve as a temporizing measure in hemodynamically unstable patients, functioning as a "bridge” to open surgery and allowing time for definitive enteric repair [1,10]. While EVAR can seal the fistulous tract, it does not eradicate the underlying fistula. Omental interposition has been identified as the strongest independent predictor of survival [4]. Recurrence of the fistula remains the most common cause of mortality (41.8%), particularly in patients who undergo simple duodenorrhaphy [4]. Although endovascular treatment can achieve effective hemorrhage control, it does not adequately address graft infection. At the time of AEF diagnosis, blood cultures should be obtained, and broad-spectrum antibiotic therapy should be initiated. In cases of positive cultures, antimicrobial treatment should be continued for four to six weeks postoperatively and adjusted based on the identified pathogens [1,3,7].

## Conclusions

AEF remains a rare but life-threatening complication, predominantly affecting elderly males with prior aortic pathology or intervention. Prompt recognition is essential, particularly in patients with a history of AAA repair presenting with gastrointestinal bleeding. CT imaging is the diagnostic modality of choice, while endoscopy is helpful for excluding other causes. Management requires a multidisciplinary approach addressing both vascular and enteric defects. Endovascular repair serves as an initial stabilization strategy, though definitive treatment often necessitates open surgical intervention. Omental interposition and infection control significantly influence survival. Early diagnosis and tailored therapy are critical to improving patient outcomes.

This case impressively demonstrates the multidisciplinary and multicenter cooperation in case of the life-threatening situation. First treatment and stabilization war performed by DCS with air transportation and definite reconstruction after arrival at the high care unit. This management may be exemplary to cope with critical incidents.
